# Cyclic diguanylate monophosphate directly binds to human siderocalin and inhibits its antibacterial activity

**DOI:** 10.1038/ncomms9330

**Published:** 2015-09-22

**Authors:** Weihui Li, Tao Cui, Lihua Hu, Ziqing Wang, Zongqiang Li, Zheng-Guo He

**Affiliations:** 1 National Key Laboratory of Agricultural Microbiology, Center for Proteomics Research, College of Life Science and Technology, Huazhong Agricultural University, Wuhan 430070, China

## Abstract

Cyclic diguanylate monophosphate (c-di-GMP) is a well-conserved second messenger in bacteria. During infection, the innate immune system can also sense c-di-GMP; however, whether bacterial pathogens utilize c-di-GMP as a weapon to fight against host defense for survival and possible mechanisms underlying this process remain poorly understood. Siderocalin (LCN2) is a key antibacterial component of the innate immune system and sequesters bacterial siderophores to prevent acquisition of iron. Here we show that c-di-GMP can directly target the human LCN2 protein to inhibit its antibacterial activity. We demonstrate that c-di-GMP specifically binds to LCN2. In addition, c-di-GMP can compete with bacterial ferric siderophores to bind LCN2. Furthermore, c-di-GMP can significantly reduce LCN2-mediated inhibition on the *in vitro* growth of *Escherichia coli*. Thus, LCN2 acts as a c-di-GMP receptor. Our findings provide insight into the mechanism by which bacteria utilize c-di-GMP to interfere with the innate immune system for survival.

## Abstract (web summary)

Siderocalin is an antibacterial component of the innate immune system that prevents iron acquisition by bacteria by sequestering their iron-binding siderophores. Here, Li *et al.* show that the bacterial second messenger c-di-GMP binds to siderocalin, thus inhibiting its antibacterial function.

## Graphical abstract

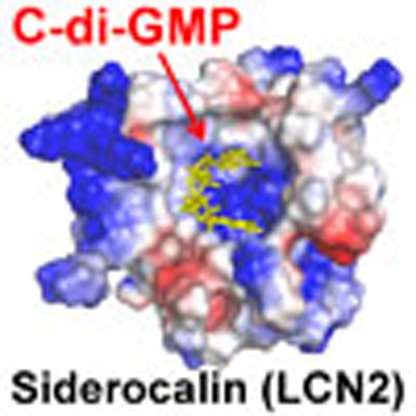

Cyclic diguanylate monophosphate (c-di-GMP) is a well-conserved second messenger found in multiple bacterial species. It participates in the regulation of a wide range of cellular processes, such as bacterial metabolism towards the sessile state/biofilm formation, motility, drug resistance and virulence^1^,^2^,^3^. c-di-GMP exhibits direct cross-talk with the innate immune system and is involved in the regulation of pathogen–host interactions. For example, c-di-GMP can bind to host immune proteins, such as STING and DDX41, and activate type I interferon response^4^,^5^. Thus, c-di-GMP can be sensed by the immune system and used to detect and eventually eliminate bacterial infection. However, studies have yet to determine whether c-di-GMP, as a conserved signaling molecule in bacteria, directly participates in resistance to host defense for bacterial survival. Siderocalin (LCN2) is a key antibacterial component of the innate immune system^6^. It belongs to the lipocalin family of immune proteins and can sequester bacterial siderophores to prevent iron acquisition and inhibit bacterial growth under iron-limited conditions^6^,^7^,^8^,^9^.

In this study, we demonstrate that c-di-GMP specifically binds to LCN2 and competes with bacterial ferric siderophores for binding the human protein. Furthermore, c-di-GMP significantly reduces LCN2-mediated inhibition on the *in vitro* growth of *Escherichia coli*. Thus, LCN2 acts as a c-di-GMP receptor and c-di-GMP can directly interact with LCN2 to inhibit its antibacterial activity.

## Results

### LCN2 is a predicted c-di-GMP receptor

**Figure 1:**
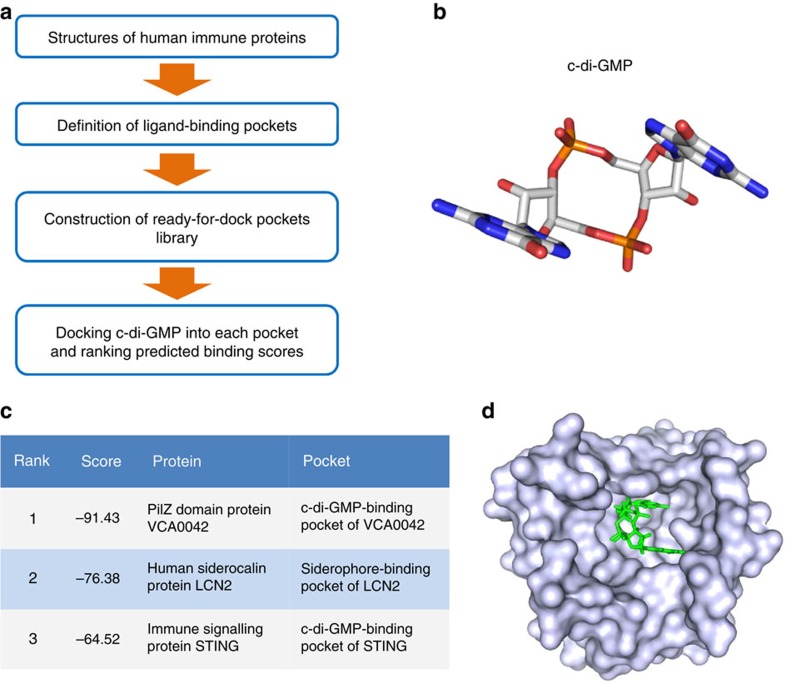
Inverse docking screen for c-di-GMP receptor from human immune proteins. (**a**) Flow diagram of c-di-GMP receptor screen from human immune proteins using the inverse docking approach. (**b**) 3D structure of the cis (or closed) c-di-GMP conformation. (**c**) Three proteins with the highest scores in the inverse docking screen. The predicted binding score of the closed c-di-GMP conformation with LCN2 is comparable to those of two known c-di-GMP receptor proteins, STING and VCA0042. (**d**) Surface representation of the siderophore-binding pockets of LCN2 in complex with c-di-GMP (green).

We first utilized an inverse docking strategy to screen candidate c-di-GMP receptors against human immune proteins, in which 1448 3D structures are currently available in the Protein Data Bank (PDB) database (Supplementary Data 1). Defining the 6.0 Å space around each ligand of protein surface, we constructed a ready-for-dock protein pocket library (Fig. 1a), then calculated and ranked the scores of the binding energy of c-di-GMP molecules (Fig. 1b, Supplementary Data 2) in each of these pockets. The 3D structure of c-di-GMP was extracted from the crystal structure of the cyclic di-GMP-VCA0042 complex, which represents a typical interaction between the c-di-GMP molecule and a receptor protein^10^. The cis (or closed) c-di-GMP conformation was further used for docking experiments and for calculating the binding energy of c-di-GMP with each protein pocket. As a characterized c-di-GMP receptor among innate immune proteins whose 3D structure has been solved^11^,^12^,^13^,^14^, STING was ranked as a potential candidate in the library and placed immediately behind VCA0042 (Fig. 1c), a reference protein used for screening and the most early characterized c-di-GMP receptor^10^. This indicates that the currently programmed inverse docking screen works well. Interestingly, LCN2 yielded a similar score as two positive proteins, namely, STING and VCA0042 (Fig. 1c). This observation suggests that siderocalin is a potential c-di-GMP receptor. Thus, our bioinformatics screen showed that LCN2 likely acts as a receptor of c-di-GMP (Fig. 1d) in addition to its function as a hunter of the ferric siderophore complex.

### c-di-GMP specifically binds to the LCN2 protein

**Figure 2:**
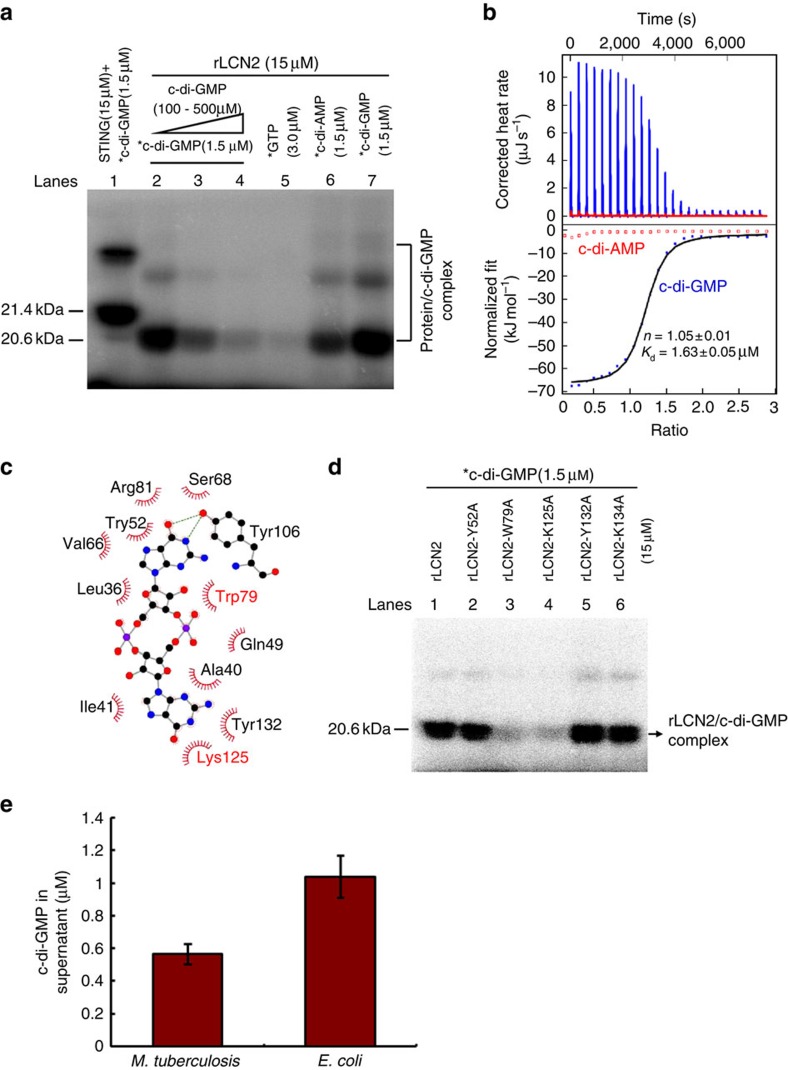
Assays for specific interaction between human LCN2 protein and c-di-GMP. (**a**) Cross-linking assay. STING was used as a positive control (lane 1). A competitive experiment was carried out by addition of unlabeled c-di-GMP at 67-, 134- and 333-fold excess to the reaction mixtures containing 15 μM rLCN2 and 1.5 μM c-di-[^32^P]GMP (lanes 2– 4). [^32^P]GTP was used as a negative control molecule. The reaction samples were subjected to a 12% w/v SDS–PAGE and radioactive gels were exposed to a storage phosphor screen (GE Healthcare). Radiolabelled nucleotides is indicated by an asterisk (*). (**b**) ITC assays for the specific interaction between rLCN2 and c-di-GMP. Original titration data and integrated heat measurements are shown in the upper and lower plots, respectively. Open and filled rectangles indicate data for c-di-AMP and c-di-GMP, respectively. The solid line in the bottom panel represents the best fit to a one-site binding model of the interaction of rLCN2 with c-di-GMP. c-di-AMP was used as a control. (**c**) Schematic of the predicted contacts between c-di-GMP and LCN2 from docking results. Potential hydrogen bonds are indicated as green dashed lines. (**d**) Cross-linking assays for the binding of c-di-GMP with five recombinant mutant LCN2 proteins. Two mutant proteins, rLCN2-W79A (lane 3) and rLCN2-K125A (lane 4), did not exhibit c-di-GMP binding activities. (**e**) Assays for c-di-GMP secretion by two bacterial strains. The concentration of extracelluar release of c-di-GMP was obtained, 1.042 μM for *E. coli* BL21(DE3) and 0.568 μM for *M. tuberculosis* H37Ra. Error bars represent the variant range of the data derived from three biological replicates.

To determine whether c-di-GMP can directly bind LCN2 protein and whether this binding is specific, we first purified the recombinant human LCN2 (rLCN2) protein without a signal peptide fragment and identified the potentially direct interaction between rLCN2 and c-di-[^32^P]GMP by performing a cross-linking assay^15^. As expected, STING, as a positive control, could clearly bind to the second messenger as indicated by an autoradiograph signal on a polyacrylamide gel electrophoresis (PAGE) gel (Fig. 2a, lane 1). We detected a similar strong signal corresponding to rLCN2 bound to c-di-[^32^P]GMP under the same conditions, which indicates that rLCN2 can directly bind to c-di-GMP (Fig. 2a, lane 7). By contrast, rLCN2 did not bind to [^32^P]GTP (Fig. 2a, lane 5) and showed very weak binding to another bacterial second messenger molecule, ^32^P-labelled cyclic diadenosine monophosphate (c-di-[^32^P]AMP; Fig. 2a, lane 6). Further experiments on c-di-[^32^P]GMP cross-linking confirmed the binding specificity of c-di-GMP in the presence of unlabeled nucleotides. Addition of unlabelled c-di-GMP at 67-, 134- and 333-fold excess to the reaction mixtures competitively inhibited the binding of rLCN2 to c-di-[^32^P]GMP (Fig. 2a, lanes 2–4). Furthermore, isothermal titration calorimetry (ITC) assays confirmed the binding specificity of c-di-GMP. Figure 2b (top) shows the raw data for titration of c-di-GMP against rLCN2 and indicates that the interaction is exothermic. Furthermore, results showed that the binding stoichiometry between c-di-GMP against rLCN2 was 1:1 (*n*=1.05), and the binding affinity of the interaction (*K*_d_) was 1.63±0.05 μM (data shown are mean±s.d. of three biological replicates) under our experimental conditions (Fig. 2b, bottom), which is comparable to the reported *K*_d_ values of several receptors^4^,^5^,^15^,^16^. By contrast, no interaction between rLCN2 and c-di-AMP (Fig. 2b) or the eukaryotic cGAMP (Supplementary Fig. 1) was detected under similar experimental conditions. These results are consistent with those of cross-linking assays. Thus, rLCN2 can directly and specifically bind to c-di-GMP. Using the ITC assay, we further performed titration of c-di-GMP into rLCN2 in a series of temperatures and calculated the heat capacity change (ΔCp) as −2.383±1.4 kJ mol^−1^ K^−1^ (data shown are mean±s.d. of three biological replicates), indicating that there exists certain conformational change^17^ for LCN2 following c-di-GMP binding.

### Two residues in LCN2 are essential for the interaction

To identify the amino acid residues involved in direct interaction between c-di-GMP and rLCN2, we introduced several point mutations to this protein and determined their effects on the binding of rLCN2 to c-di-GMP. Based on docking analysis, several amino acid residues in the putative binding pocket of rLCN2 were predicted to interact directly with c-di-GMP (Fig. 2c). Five recombinant proteins with single residue mutations, including rLCN2-Y52A, rLCN2-W79A, rLCN2-K125A, rLCN2-Y132A and rLCN2-K134A, were expressed and successfully purified. We further performed c-di-[^32^P]GMP cross-linking assays to examine the importance of these residues for binding of c-di-GMP. Figure 2d shows that the protein lost its c-di-[^32^P]GMP binding activity when either residue Trp79 or Lys125 was mutated, as indicated by lack of specific autoradiograph band on a PAGE gel (Fig. 2d, lanes 3 and 4). By contrast, three other mutant proteins, rLCN2-Y52A, rLCN2-Y132A and rLCN2-K134A, retained the c-di-[^32^P]GMP binding ability of the wild-type rLCN2 protein (Fig. 2d). These results suggest that the Trp79 and Lys125 residues are essential for specific interaction between c-di-GMP and rLCN2, and that c-di-GMP can directly bind to rLCN2.

### c-di-GMP can be secreted by bacteria

c-di-GMP binds to the same pocket as the ferric siderophore complex on LCN2. This suggests that c-di-GMP may compete with ferric siderophore in binding to siderophore, and thereby interfere with its antibacterial activity. We first confirmed that c-di-GMP can be secreted by the fast-growing bacteria *E. coli* and the slow-growing species *Mycobacterium tuberculosis*, which is a human pathogen that causes tuberculosis (TB)^18^. Using liquid chromatography and mass spectrometry (LC–MS), we determined the concentrations of c-di-GMP in bacterial culture supernatants to be 1.042±0.12 μM for *E. coli* cells and 0.568±0.06 μM for *M. tuberculosis* (Fig. 2e; data shown are mean±s.d. of three biological replicates). However, no c-di-GMP was detected from the control medium. Therefore, c-di-GMP can be secreted by both *E. coli* and *M. tuberculosis.*

### c-di-GMP can compete with carboxymycobactin

**Figure 3:**
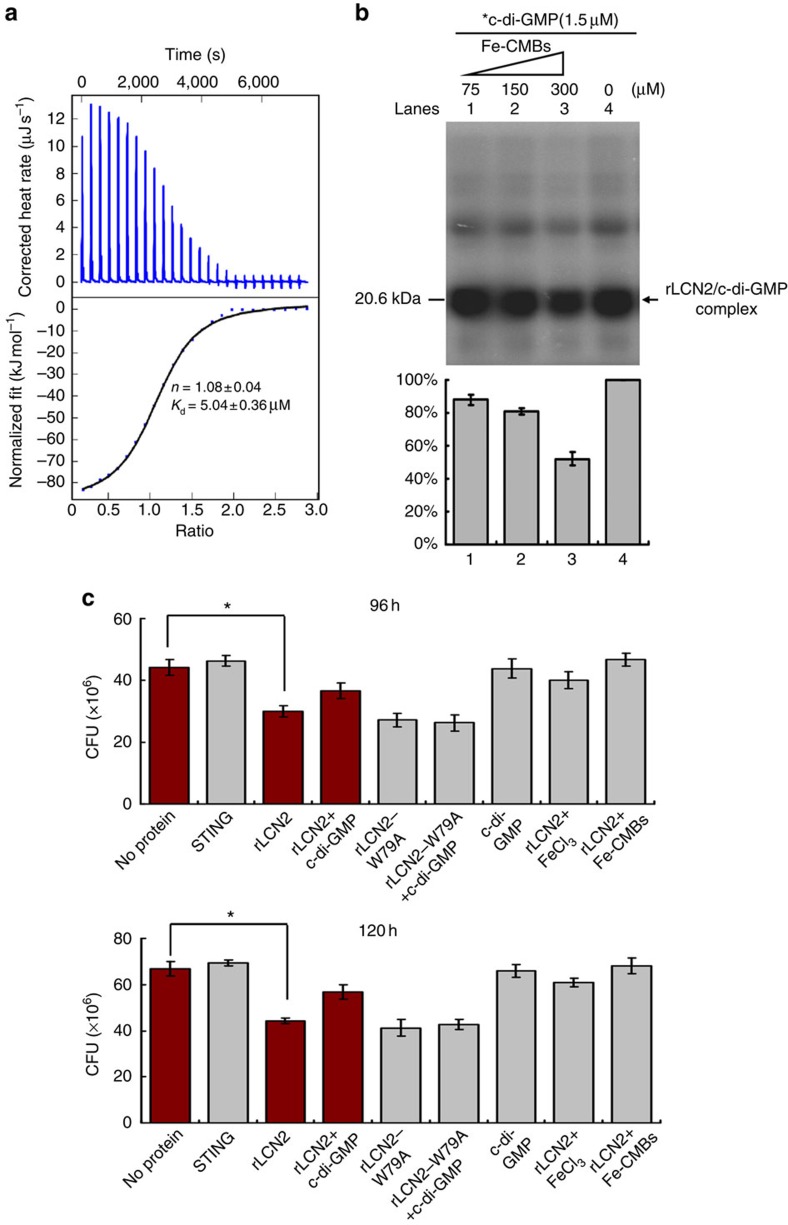
Assays for rLCN2-mediated inhibition of *M. tuberculosis* growth in the presence or absence of c-di-GMP. (**a**) ITC assays for the interaction between Fe-CMBs and rLCN2. Original titration data and integrated heat measurements are shown in the upper and lower plots, respectively. (**b**) Cross-linking assay for the ability of c-di-[^32^P]GMP to bind to rLCN2 with Fe-CMBs. c-di-[^32^P]GMP retained >50% of the rLCN2 binding capacity even in the presence of 200-fold excess of Fe-CMBs (lane 3). The reaction samples were assayed on a 12% w/v SDS–PAGE. The c-di-[^32^P]GMP-rLCN2 complex was quantified, and the mean values of three independent labeling experiments along with error bars are shown. (**c**) Assays for changes in *M. tuberculosis* H37Ra growth mediated by the human rLCN2 protein in the presence or absence of c-di-GMP. FeCl_3_ and Fe-CMBs were used as controls for the growth inhibition assays. Bacterial counts were determined at two representative time points, 96 h and 120 h. and an asterisk (*) represents significant difference (*P*≤0.05, two-tailed Student's *t*-test) between two groups.

In an ITC assay, we found that both rLCN2 (5.04±0.36) (Fig. 3a) and the mutant protein rLCN2-W79A (4.56±0.47 ) (data shown are mean±s.d. of three biological replicates; Supplementary Fig. 2a) could bind well to ferric carboxymycobactins (Fe-CMBs), although the mutant protein rLCN2-W79A almost lost c-di-GMP binding activity (Supplementary Fig. 2b). This observation indicated that some amino acid residues recognized by c-di-GMP may be different from those recognized by siderophores, even though the two molecules bind to the same pocket on LCN2. Using a similar c-di-[^32^P]GMP cross-linking assay (Fig. 2d), we further determined the competitive binding activity of c-di-[^32^P]GMP to the protein in the presence of Fe-CMBs. c-di-[^32^P]GMP retained a clear rLCN2 binding capacity even in the presence of 200-fold excess of Fe-CMBs (Fig. 3b, lane 3). This result indicated that c-di-[^32^P]GMP could easily compete with ferric carboxymycobactin for protein binding.

### c-di-GMP reduces LCN2-mediated inhibition of *M. tuberculosis*

Next, we investigated whether c-di-GMP can target human LCN2 and contribute to the survival of *M. tuberculosis*. LCN2 exhibits similar ability to inhibit *in vitro* growth of several mycobacterial strains, including *M. tuberculosis* H37Rv, *M. tuberculosis* H37Ra and *M. bovis* Bacillus Calmette Guérin (BCG)^19^. We used *M. tuberculosis* H37Ra as a model to further evaluate the effect of c-di-GMP on rLCN2-mediated mycobacterial growth inhibition under iron-limiting conditions. As shown in Fig. 3c, STING, a negative control protein that cannot bind ferric carboxymycobactin (Supplementary Fig. 3a), did not inhibit the growth of *M. tuberculosis*. By contrast, both rLCN2 and the mutant rLCN2-W79A could inhibit the growth of *M. tuberculosis* under the same conditions (Fig. 3c). However, the inhibitory effect of rLCN2 was limited (Fig. 3c). This result is similar to those reported in previous studies^20^ and may be explained by the extremely slow growth rate of *M. tuberculosis* and loss of rLCN2 activity with extended culture time. Under such conditions, stable c-di-GMP-protein complexes may not form easily. This is consistent with our observation that c-di-GMP could partially, but not significantly, rescue rLCN2-mediated growth inhibition (Fig. 3c). Furthermore, c-di-GMP alone did not affect bacterial growth under similar conditions (Fig. 3c). Taken together, these results suggest that c-di-GMP competes with carboxymycobactin for binding to LCN2, thereby reducing LCN2-mediated growth inhibition of *M. tuberculosis*.

### c-di-GMP rescues rLCN2-mediated inhibition of *E. coli* growth

**Figure 4:**
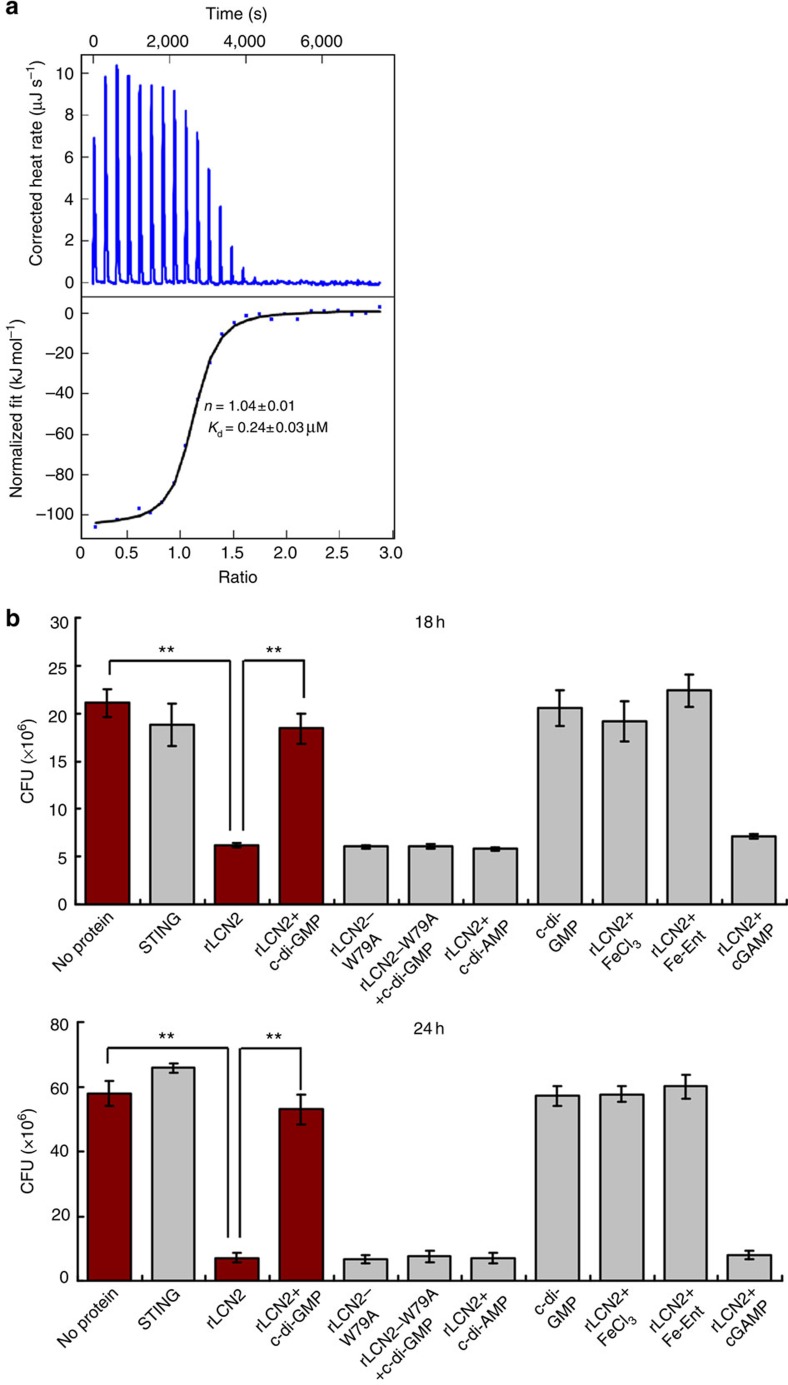
Assays for LCN2-mediated inhibition of *E. coli* growth in the presence or absence of c-di-GMP. (**a**) ITC assays for the interaction between Fe-Ent and rLCN2 protein. Original titration data and integrated heat measurements are shown in the upper and lower plots, respectively. (**b**) Assays for changes in *E. coli* growth mediated by the human rLCN2 protein in the presence or absence of c-di-GMP. c-di-GMP could rescue the growth inhibition by rLCN2, but hardly by rLCN2-W79A. Addition of FeCl_3_ or Fe-Ent could rescue rLCN2-mediated growth inhibition. The control nucleotide, c-di-AMP or cGAMP, could not rescue rLCN2-mediated growth inhibition. Bacterial counts were determined at two representative time points, 18 h (top panel) and 24 h (bottom panel). Double asterisks (**) represent significant difference between two groups (*P*≤0.01, two-tailed Student's *t*-test).

Next, we used *E. coli*, a fast-growing bacterium, as a model to determine whether rLCN2-mediated bacterial growth inhibition is rescued by c-di-GMP. rLCN2-mediated inhibition of growth has been previously described in *E. coli*^7^,^8^. In a similar ITC assay, we found that the rLCN2 protein could bind well to ferric enterobactin (Fe-Ent; Fig. 4a). The binding affinity of the interaction (*K*_d_) was 0.24±0.03 μM (data shown are mean±s.d. of three biological replicates). The mutant rLCN2-W79A protein without the ability to bind c-di-GMP retained good binding activity for Fe-Ent (*K*_d_ 0.25±0.05 μM) (data shown are mean±s.d. of three biological replicates) (Supplementary Fig. 3b), which is comparable to the wild-type protein. Using a c-di-[^32^P]GMP cross-linking assay (Supplementary Fig. 4), we determined the competitive binding activity of c-di-[^32^P]GMP to the protein in the presence of Fe-Ent. Interestingly, binding of c-di-[^32^P]GMP to rLCN2 could be hardly detected in the presence of 100-fold excess Fe-Ent (Supplementary Fig. 4, lane 6). This result indicated that Fe-Ent could easily compete with c-di-[^32^P]GMP for protein binding.

We further analysed the antibacterial activity of rLCN2 and the effect of c-di-GMP on rLCN2-mediated growth of *E. coli* under iron-limiting conditions. As shown in Fig. 4b, 0.15 μM rLCN2 protein inhibited the growth of *E. coli* in an iron-depleted M9 broth. By contrast, the STING protein, which is incapable of binding Fe-Ent (Supplementary Fig. 5), did not significantly interfere with growth of *E. coli* under similar conditions. This result suggested that the currently rLCN2-mediated bacterial growth inhibition worked well. Furthermore, growth inhibition by rLCN2 was clearly rescued when 7.5 μM c-di-GMP, together with rLCN2, were added to the medium (Fig. 4b). However, c-di-GMP did not significantly rescue the mutant rLCN2-W79A protein-mediated growth inhibition (Fig. 4b). Furthermore, the control nucleotide, c-di-AMP or cGAMP, could not rescue rLCN2-mediated growth inhibition. Thus, c-di-GMP could specifically inhibit rLCN2-mediated inhibition of *E. coli* growth under iron-limiting conditions.

## Discussion

As a well-known signalling molecule, the functions of c-di-GMP have been extensively characterized in bacteria. However, the mechanism by which the second messenger directly interacts with the host innate immune system is only partially understood. Thus far, STING and DDX41 are the only two identified c-di-GMP receptor proteins in the host immune system that activate innate immune response and contribute to successful pathogen elimination^4^,^5^. However, *M. tuberculosis* is known to be an intracellular pathogen and can survive in the host cells, indicating that bacteria can employ alternative strategies to escape the host defense system. Using a computational inverse docking strategy, we identified LCN2, a key antibacterial protein, as a new c-di-GMP receptor. This result marks the identification of a new pathogen–host interaction. We confirmed that c-di-GMP directly interacted with LCN2 and competed with bacterial siderophores to bind to the protein. Furthermore, we found that the bacterial second messenger prevented LCN2 from inhibiting bacterial growth. Taken together, these results indicate that c-di-GMP would participate in resistance to host defense by interfering with the innate immune protein LCN2 to ensure bacterial survival during infection. Meanwhile, the c-di-GMP-LCN2 interaction would represent a novel PAMP/PRR (pathogen-associated molecular pattern/pathogen recognition receptor) sensing mechanism that may trigger a host immune response. However, it requires further experiment evidences.

LCN2 does not share sequence homology with known receptor proteins. As such, LCN2 represents a novel category of c-di-GMP receptors. LCN2 specifically recognizes c-di-GMP but not c-di-AMP. This observation is clearly different from the case of the two identified host proteins, STING and DDX41, that recognize both c-di-GMP and c-di-AMP^4^,^5^. In addition, the binding mode of c-di-GMP to LCN2 is also different. Structural reports have shown that one molecule of c-di-GMP binds to one dimer of STING^11^. On the basis of the results of an inverse docking screen and point-mutation analysis, we propose that one molecule of c-di-GMP binds to one LCN2 monomer in the siderophore-binding pocket. The mechanism by which c-di-GMP binds to LCN2, as shown by co-crystallization studies, requires further analysis.

Iron is essential for pathogens to establish infection in hosts. Several bacterial species can acquire iron from an iron-limiting host environment by secreting siderophores. However, hosts have evolved a specific iron-depletion strategy to arrest bacterial siderophores via LCN2, a key innate immune protein^6^,^7^,^8^,^9^. During infection, LCN2 can intercept the ferric siderophore complex and inhibit bacterial growth. Therefore, iron acquisition pathways are attractive targets because they are essential for the survival of intracellular pathogenic bacteria and establishment of infection in host environments^21^. As such, efforts have been devoted to the development of inhibitors targeting the siderophore biosynthesis pathway^22^,^23^. Our current findings indicate that c-di-GMP signaling also mediates important functions in siderophore-mediated iron acquisition pathways in pathogenic bacteria. As a pathogen becomes increasingly susceptible to LCN2, inhibition of production or extracellular release of c-di-GMP should attenuate its virulence. Thus, c-di-GMP signalling may be an effective alternative target to block bacterial iron acquisition and thereby prevent or stop bacterial infection.

## Methods

### Reagents

c-di-GMP, c-di-AMP and cGAMP used for bacterial growth inhibition assays were purchased from BIOLOG Life Science Institute (Germany). Fe-CMBs and Fe-Ent were purchased from Biophore Research Products (Germany), thrombin was sourced from Biosharp (China) and glutathione-sepharose 4B column was purchased from GE Healthcare. Both radioactive-labelled [α-^32^P]GTP and [α-^32^P]ATP were purchased from Perkin Elmer (USA). Middlebrook 7H9 medium and Oleic acid-Albumin-Dextrose-Catalase (OADC) enrichment were sourced from Becton-Dickinson Company (USA) and were used for growing *M. tuberculosis*. GTP, dNTP and DNA polymerase for PCR assays were obtained from TaKaRa Co. (Japan). Both pET28a and pGEX-4 T-1vectors, which were used for expressing rLCN2 proteins. All restriction enzymes for cloning were obtained from TaKaRa Co.

### Inversing docking screen

A total of 1448 3D structures of 167 proteins (including STING) involved in human immune system were searched and downloaded from the PDB database (http://www.rcsb.org)^24^ with the following criteria: Keywords: ‘immune', Taxonomy: ‘just *Homo sapiens* (human)' and Experimental Method: ‘X-RAY'. The ligands in structures were then identified by parsing ‘HET' records. Several kinds of ligands, including solvent molecules, modified residues, anions and ligands whose atom numbers were <5 or >130, were filtered. Finally, 751 ligands were identified from 1,448 structures. A 6.0 Å space around each ligand was defined as the candidate-binding pocket. The PDB IDs of 1448 structures, 751 pockets and 166 proteins can be found in Supplementary Data 1–3. Two known c-di-GMP receptor proteins, namely, VCA0042 (ref. 10) and STING^11^, were added to the protein library as positive controls.

Following the DOCK 6 protocol, each protein structure was processed according to protocol recommended in official documents of DOCK (http://dock.compbio. ucsf.edu/DOCK_6/tutorials/index.htm). Hydrogen was added to generate protonation states at physiological pH by the chimera tool *AddH*. Charges for standard amino acids were assigned from Amber using the chimera tool *Add Charge*. Histidine side chains were protonated based on their local environment. Side chains of other residue types were assigned protonation states at physiological pH (http://www.cgl.ucsf. edu/chimera /current/docs/ContributedSoftware/addh/addh.html). Molecular surface data of each structure were calculated using the chimera tools Surface and Write DMS. The box encompassing the selected spheres was generated using the DOCK suite program showbox. Grid files necessary for rapid score evaluation in DOCK were calculated with the DOCK suite program grid. Thus, a ready-for-dock protein pocket library containing 751 pockets (Supplementary Data 3) was constructed.

The 3D structure of c-di-GMP was extracted from the c-di-GMP-VCA0042 structure complex (PDB ID: 2RDE, chain identifier: A, residue name: C2E, residue sequence number: 301)^10^, and further integrated with hydrogen atoms and charge information. c-di-GMP ligand was assigned protonation states at physiological pH using the chimera tool AddH. Charges were assigned following Austin Model 1 with Bond Charge Correction (AM1-BCC) charge calculation methods using the chimera tool *Add Charge*.

The score for binding energy of c-di-GMP with each protein was calculated and evaluated one-by-one using DOCK 6 (ref. 25). The scores corresponding to each protein in the constructed library were ranked and listed.

### Protein-ligand contact analysis and visualization

Structural figures were prepared using Chimera^26^ and pymol (http:// http://www.pymol.org/). Protein-ligand interactions were analyzed using LigPlot^+^ (ref. 27).

### Expression and purification of rLCN2

Construction of expression plasmid for the *rLCN2* gene and purification of glutathione *S*-transferase (GST)-fused proteins were performed as described previously with modifications^20^. A pair of primers (5′-ACGA GAATTC AA ATGCAGGACTCCACCTCAGACCT-3′ and 5′-ATCA TCTAGA TCAGCCGTCGATACACTGGTCGA-3′) was used to amplify the *LCN2* gene lacking signal peptide sequence by PCR. The amplification was performed as follows: 95 °C for 5 min, followed by 30 cycles of 95 °C for 1 min, 50 °C for 30 s, 72 °C for 1 min and a final extension of 72 °C for 8 min, respectively. The PCR products were digested by restriction endonucleases and then inserted into the modified pGEX-4 T-1 vector. Several mutant *rLCN2* genes were produced by site-directed mutagenesis and overlap extension PCR. Five pairs of mutagenic PCR primers were designed, in which 5′-AAGACCCGCAAAAGATGGCTGCCACCAT CTATGAGC-3′ and 5′-GCTCATAGATGGTGGCAGCCATCTTTTGCGGGTCTT-3′ for amplification of rLCN2-Y52A, 5′-AAAAGAAGTGTGACTACGCGATCAGGA CTTTTGTTC-3′ and 5′-GAACAAAAGTCCTGATCGCGTAGTCACACTTCTTT T-3′ for amplification of rLCN2- W79A, 5′-CTATGGTGTTCTTCAAGGCAGTT TCTCAAA ACAGG-3′ and 5′-CCTGTTTTGAGAAACTGCCTTGAAGAACACC ATAG-3′ for amplification of rLCN2- K125A, 5′-TTTCTCAAAACAGGGAGGCC TTCAAGA TCACCCTCT-3′ and 5′-AGAGGGTGATCTTGAAGGCCTCCCTGT TTTGAGAA A-3′ for amplification of rLCN2- Y132A, 5′-AAAACAGGGAGTAC TTCGCGATCACCCTCTACGGGA-3′ and 5′-TCCCGTAGAGGGTGATCGCGAAG TACTCCCTGTTT T-3′ for amplification of rLCN2-K134A. The recombinant plasmids containing wild-type or mutant *rLCN2* gene were transformed into *E. coli* BL21 (TaKaRa Company), respectively, to produce expression strains. Then they were grown at 37 °C with an optical density of up to 0.9 at 600 nm and protein expression was induced for 3–4 h by addition of 0.5 mM isopropyl β-D-1-thiogalactopyranoside. Harvested cells were resuspended and sonicated. The cell lysate was centrifuged at 10,000*g* for 30 min, and the clear supernatant was loaded into a GST-affinity purification column. The column-bound protein was washed with PBS buffer. The protein was then eluted using PBS buffer containing 20 mM reduced glutathione. The purified proteins were incubated with thrombin (Biosharp) to cleave the GST tag, and were further purified with glutathione-sepharose 4B (GE Healthcare). The human *STING* gene was amplified by PCR using a pair of primers (5′-ATATGAATTCAAATGGTGG CCCATGG GCTGGCATG-3′ and 5′-CGCG TCTAGATCAAACCTCTTCCTTTT CCT CCT-3′). Then the STING protein was expressed and purified according to the procedure described previously^4^. The purified proteins were resolved by 12% SDS–PAGE. Protein concentration was detected using the Bradford method.

### Cross-linking assays for nucleotide-LCN2 interaction

Radioactive-labeled c-di-GMP was enzymatically produced from [α-^32^P]GTP (3000 Ci mmol^−1^) by *E. coli* diguanylate cyclase encoded by *ydeH* gene^28^. The gene fragments were amplified by PCR using a pair of primers (5′-GCGC GAATTC AAATGATCAA GAAGACAACG-3′ and 5′-GGCC TCTAGA TTAAACT CGGTTAATCAC-3′). After digested by restriction endonucleases, PCR products were inserted into pET28a vector. The YdeH proteins were then purified on Ni^2+^ affinity columns. c-di-[^32^P]AMP was produced from [α-^32^P]ATP by the *Bacillus subtilis* DisA protein, which has an adenylate cyclase activity^29^. The *disA* gene fragments were amplified by PCR using a pair of primers (5′-AGAG GAATTC TG ATGGAAAAAGAGAAAAAAGG-3′ and 5′-AGAG TCTAGA TCACAGTTG TCTGTCTAAATAA -3′) and then cloned into pET28a vector, and the 6 × His-DisA proteins were purified on Ni^2+^ affinity columns. To produce radioactive-labelled c-di-GMP or c-di-AMP, 10 μM YdeH or DisA proteins were added into the reaction buffer containing 50 mM Tris-HCl, pH 7.5, 50 mM NaCl and 5 mM MgCl_2_. The reactions were started by the addition of 30 μM [α-^32^P]GTP or [α-^32^P]ATP. After running overnight at 37 °C, the reactions were stopped by placing it in boiling water for 10 min. The produced c-di-[^32^P]GMP or c-di-[^32^P]AMP was further purified by centrifugation and filtration. Their concentrations were determined by thin-layer chromatography and stored at −20 °C until for use. For cross-linking assay, 15 μM rLCN2 proteins were incubated for 10 min on ice in diguanylate cyclase reaction buffer (50 mM Tris-HCl, pH 7.5, 50 mM NaCl, 5 mM MgCl_2_) together with 1.5 μM radioactive-labelled c-di-GMP, c-di-AMP or GTP. And the competing cold ligands c-di-GMP, Fe-CMBs and Fe-Ent were added at the same time with radioactive-labelled c-di-GMP. Following ultraviolet irradiation for 30 min, reaction samples were directly subjected to a 12% w/v SDS–PAGE. Electrophoresis was performed at 150 V and at room temperature until bromophenol blue reached the bottom part of the gel. Radioactive gels were exposed to a storage phosphor screen (GE Healthcare) overnight. Images were obtained, and the protein-c-di-GMP complex was quantified using Typhoon Scanner (GE healthcare).

### ITC analysis

ITC measurements were carried out at 25 °C with a Nano ITC Low Volume isothermal calorimeter (TA Instruments, New Castle, DE) controlled by ITCRun software. All proteins were dialyzed against the same buffer (20 mM Tris base, 100 mM NaCl, 5 mM MgCl_2_, pH 7.5) and all buffers were degassed before use. The protein (60 μM) and the c-di-GMP/Fe-CMBs/Fe-Ent solution (500 μM) were added to the sample cell (190 μl) and the syringe (50 μl), respectively. There were 25 injections per experiment, and the stirring rate was 250 r.p.m. Data were recorded automatically and subsequently analyzed using the NanoAnalyze Software provided by the manufacturer. In control experiments, the c-di-GMP/Fe-CMBs/Fe-Ent solution was titrated into the buffer in sample cells to obtain the heat of dilution. The value of the heat of dilution was then subtracted from the experimental curve in the final analysis (Supplementary Table 1). All the titration curves were fitted to the independent-site binding model.

### Detection of c-di-GMP secreted by bacteria

The strains of *E. coli* BL21(DE3) and *M. tuberculosis* H37Ra were cultured with shaking at 37 °C until OD_600_=0.5 in 1000, ml Luria-Bertani (LB) medium containing 10 g NaCl, 10 g tryptone and 5 g yeast extract (pH 7.0) and 7H9 media containing 0.5 g ammonium sulfate, 0.5 g L-glutamic acid, 0.1 g sodium citrate, 1.0 mg pyridoxine, 0.5 mg biotin, 2.5 g disodium phosphate, 1.0 g monopotassium phosphate, 0.04 g ferric ammonium citrate, 0.05 g magnesium sulfate, 0.5 mg calcium chloride, 1.0 mg zinc sulfate and 1.0 mg copper sulfate, respectively. The cultures were harvested by centrifugation. Bacterial cell pellets were vacuum-freeze dried and then weighed. The supernatants were subsequently filtered through 0.22 μm nitrocellulose membrane filter and were concentrated by vacuum freeze-drying to 6.8 ml for *E. coli* and 6.25 ml for *M. tuberculosis*, respectively.

The supernatant samples (15 μl) were injected into C-18 columns for HPLC analysis and separated by reverse-phase HPLC (Waters e2695). Buffer A (100 mM KH_2_PO_4_, pH 5.9) and buffer B (75% (v/v) buffer A and 25% (v/v) methanol) were used at a 2:1 gradient at a flow rate of 0.7 ml min^−1^. Nucleotides were detected at 254 nm wavelength. The samples were analyzed using an Agilent series liquid chromatograph (Agilent, USUXK02634) connected in-line with an TOF/Q-TOF mass spectrometer equipped (Agilent, G6540A) with an electrospray ionization source (Dual ESI). The LC was equipped with C18 guard (ZORBAX Eclips Plus C18, 2.1 × 100 mm, 3.5 μm). Eluent B consisted of 10 mM ammonium acetate and 0.1% (v/v) acetic acid in water and eluent A was methanol. The injection volume was 50 μl and the flow rate was 0.4 ml min^−1^ throughout the chromatographic run. Hundred percent B was used from 0 to 6.3 min followed by a linear gradient from 100% B to 90% B until 8.3 min, a linear gradient from 90% B to 70% B until 11 min and a linear gradient from70% B to 10% B until 12 min. 10% B was used from 12 to 14 min and 99% B was used from 14.1 to 20 min. The analyte detection was performed on an TOF/Q-TOF mass spectrometer equipped (Agilent, G6540A) with an electrospray ionization source (Dual ESI)^30^. The observed peak for ion 691.10 m/z was integrated using Xcalibur software. The concentration of the nucleotide was determined by comparing the integrated peak area to a calibration curve generated using the purified c-di-GMP positive control samples of known concentration analyzed in a similar way. Error bars represent the variant range of the data derived from three biological replicates.

### c-di-GMP rescue experiments

For *in vitro* growth measurements, the strains of *M. tuberculosis* H37Ra were cultured at 37 °C in 1000, ml Middlebrook 7H9-OADC medium containing 0.5 g ammonium sulfate, 0.5 g L-glutamic acid, 0.1 g sodium citrate, 1.0 mg pyridoxine, 0.5 mg biotin, 2.5 g disodium phosphate, 1.0 g monopotassium phosphate, 0.04 g ferric ammonium citrate, 0.05 g magnesium sulfate, 0.5 mg calcium chloride, 1.0 mg zinc sulfate, 1.0 mg copper sulfate, 0.85 g sodium chloride, 5 g bovine albumin (Fractionv), 2 g dextrose and 3 mg catalase. Subsequently, 10^5^–10^6^ Colony-Forming Units (CFU) of *M. tuberculosis* H37Ra were incubated in 4 ml Middlebrook 7H9-OADC medium with or without 0.3 μM rLCN2 for 96 h and 120 h at 37 °C. The growth was measured as CFU from serial dilutions plated onto Middlebrook 7H10-OADC agar plates (1000, ml medium containing 0.5 g ammonium sulfate, 1.5 g monopotassium phosphate, 1.5 g disodium phosphate, 0.4 g sodium citrate, 25.0 mg magnesium sulfate, 0.5 mg calcium chloride, 1.0 mg zinc sulfate, 1.0 mg copper sulfate, 0.5 g L-glutamic acid (sodium salt), 0.04 g ferric ammonium citrate, 1.0 mg pyridoxine hydrochloride, 0.5 mg biotin, 250.0 μg malachite green, 0.85 g sodium chloride, 2 g dextrose, 5 g bovine albumin (Fractionv), 3 mg catalase and 0.06 ml oleic acid and 15 g agar) and incubated at 37 °C for 25 days. The strains of *E. coli* BL21(DE3) were cultured to log phase shaking at 37 °C in M9 medium (1000, ml M9 medium containing 12.8 g Na_2_HPO_4_.7H_2_O, 3 g KH_2_PO_4_, 0.5 g NaCl, 1 g NH_4_Cl, 2 ml of 1 mol l^−1^ MgSO_4_, 20 ml of 20% glucose and 0.1 ml of 1 mol l^−1^ CaCl_2_). Subsequently, 10^3^–10^4^ CFU of log phase *E. coli* BL21(DE3) were incubated in 3 ml M9 medium with or without 0.15 μM rLCN2 for 18 h and 24 h at 37 °C. The growth was measured as CFU from serial dilutions plated onto LB agar plates (1000, ml medium containing 10 g NaCl, 10 g tryptone, 5 g yeast extract and 15 g Agar, pH 7.0 ) and incubated at 37° for 18 h. To determine the effect of c-di-GMP on rLCN2-mediated growth inhibition of *E. coli*, we pre-incubated c-di-GMP together with wild-type or mutant rLCN2 protein on ice for 30 min, then added it into the medium (7.5 μM). To determine whether it had a similar effect on *M. tuberculosis* H37Ra, a final concentration of 9 μM c-di-GMP was added into the medium containing 0.3 μM rLCN2. The human STING protein (0.3 μM) was used as positive control. Both c-di-AMP and cGAMP were used nucleotide controls for growth inhibition experiments of *E. coli*. Error bars represent the variant range of the bacterial growth data derived from three biological replicates. The *P*-values of the relative growth data were calculated by unpaired two-tailed Student's *t*-test using GraphPad Prism 5.

## Additional information

**How to cite this article:** Li, W. *et al.* Cyclic diguanylate monophosphate directly binds to human siderocalin and inhibits its antibacterial activity. *Nat. Commun.* 6:8330 doi: 10.1038/ncomms9330 (2015).

## Supplementary Material

Supplementary Information Supplementary Figures 1-5 and Supplementary Table 1

Supplementary Data 1 The PDB ids list of 3D structures potentially involved in human immune system.

Supplementary Data 2 Scoring list of c-di-GMP with non-redundant proteins (including VCA0042) and their pockets.

Supplementary Data 3 The identified pockets from structures and corresponding proteins.
