# Supplementary material for: Cyclic diguanylate monophosphate directly binds to human siderocalin and inhibits its antibacterial activity
Source: Nat Commun. 2015 Sep 22;6:8330. doi: 10.1038/ncomms9330 (PMC4595737; doi:10.1038/ncomms9330)
Supplement: Supplementary Information — Supplementary Figures 1-5 and Supplementary Table 1 [file ncomms9330-s1.pdf]

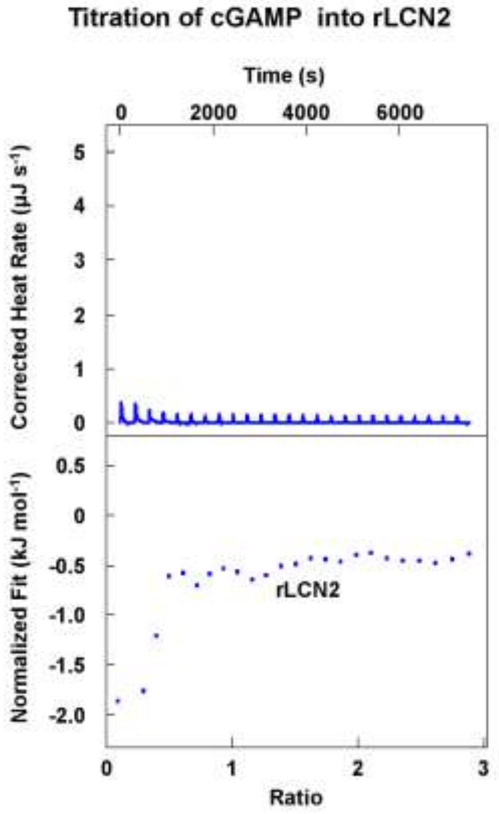

Supplementary Figure 1. ITC assay for the interaction between cGAMP and rLCN2. Original titration data and integrated heat measurements are shown in the upper and lower plots, respectively.

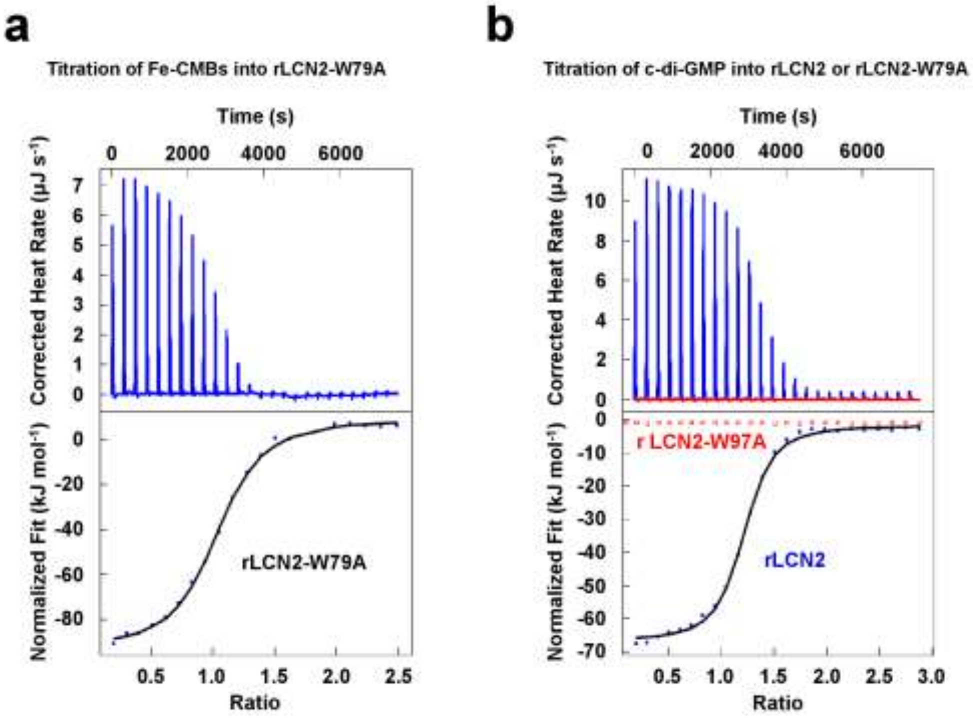

Supplementary Figure 2. ITC assays for the interaction between the mutant protein rLCN2-W79A and Fe-CMBs or c-di-GMP. (a) The interaction between Fe-CMBs and rLCN2-W79A protein. **(b)** The interaction between c-di-GMP and rLCN2 /rLCN2-W79A protein. Original titration data and integrated heat measurements are shown in the upper and lower plots, respectively. Blue and red indicate data for rLCN2 and rLCN2-W79A, respectively.

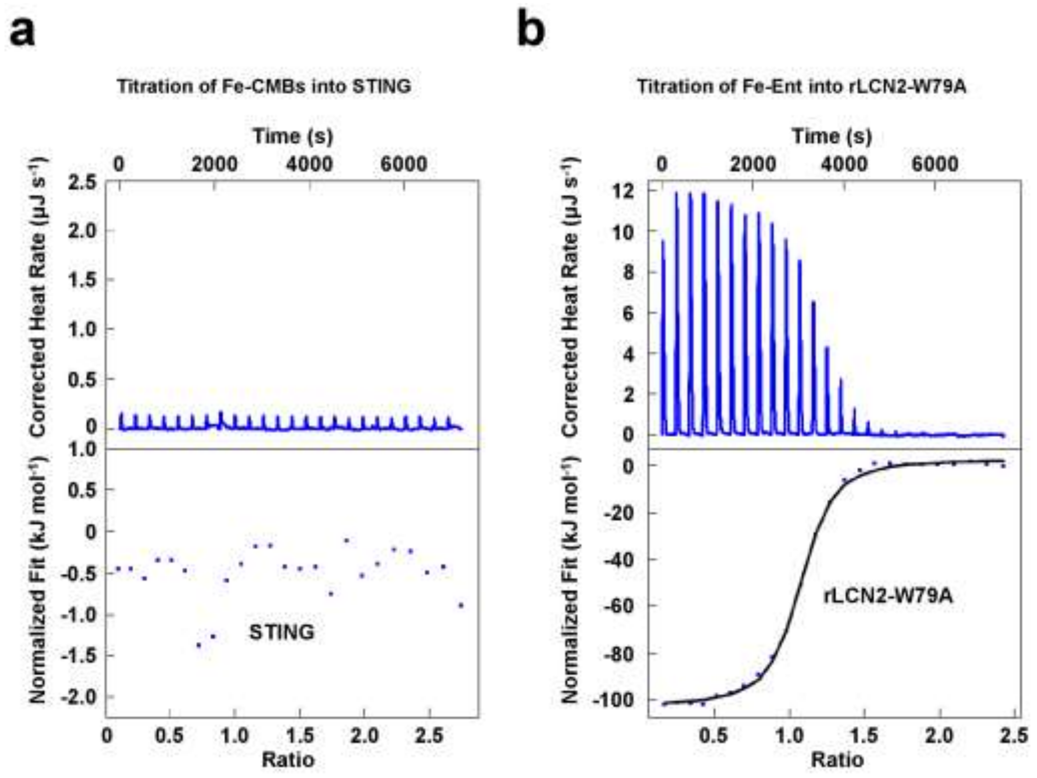

Supplementary Figure 3. ITC assays for the interaction between ferric siderophores and STING or the mutant protein rLCN2-W79A. (a) The interaction between Fe-CMBs and STING protein. **(b)** The interaction between Fe-Ent and rLCN2-W79A protein. Original titration data and integrated heat measurements are shown in the upper and lower plots, respectively.

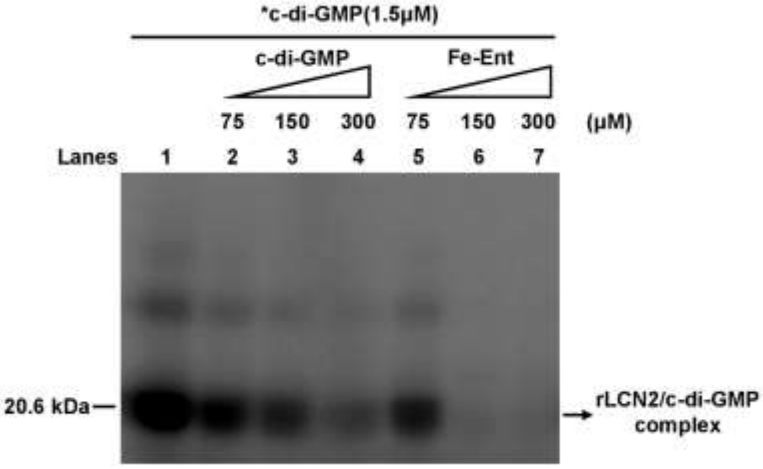

Supplementary Figure 4. Cross-linking assay for the ability of c-di-[^{32}P]GMP to bind to rLCN2 with Fe-Ent. Competitive experiments were carried out by addition 75-300 μM of unlabeled c-di-GMP (lanes 2 to 4) or Fe-Ent (lanes 5 to 7) to the reaction mixtures containing 15 μM rLCN2 and 1.5 μM c-di-[^{32}P]GMP (lanes 2 to 4). The reaction samples were assayed on a 12% w/v SDS–PAGE.

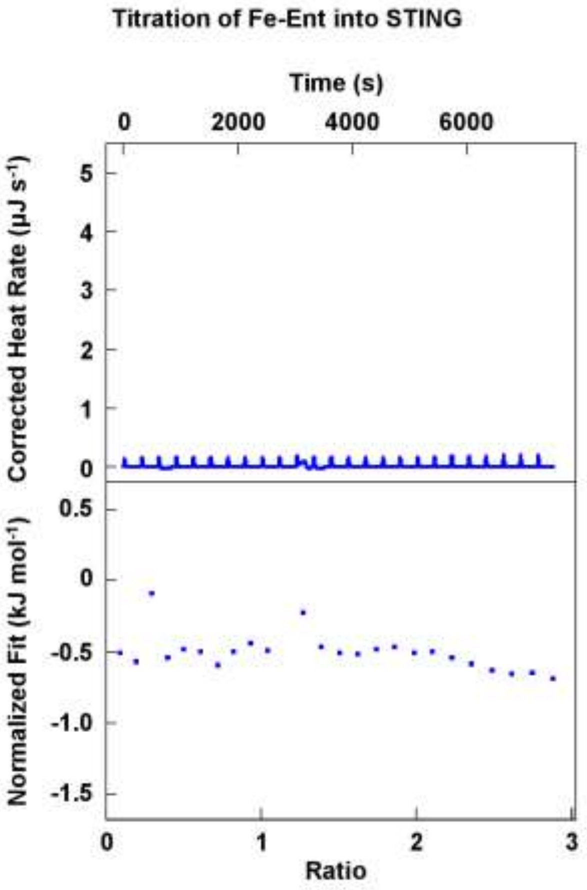

Supplementary Figure 5. ITC assay for the interaction between Fe-Ent and STING. Original titration data and integrated heat measurements are shown in the upper and lower plots, respectively.

Supplementary Table 1. Thermodynamic parameters measured by ITC at 25 ℃

	LCN2				LCN2-W97A			
	ΔG kJ mol^{-1}	ΔH kJ mol^{-1}	ΔS J mol^{-1} K^{-1}	*n*	ΔG kJ mol^{-1}	ΔH kJ mol^{-1}	ΔS J mol^{-1} K^{-1}	*n*
Fe-CMBs	-27.81±1.05	-91.77±2.26	-214.5±3.15	1.079 ±0.04	-30.81±2.05	-102.19±3.12	-239.4±3.04	0.996 ±0.04
Fe-Ent	-31.8±1.89	-106.67±2.62	-251.1±3.22	1.039 ±0.01	-30.9±1.14	-105.5±4.94	-250.2±3.11	1.041±0.01
c-di-GMP	-31.16±1.52	-65.39 ±1.36	-114.8 ±2.05	1.045±0.01	/	/	/	/
